# Ultrasonic-assisted preparation of two-dimensional materials for electrocatalysts

**DOI:** 10.1016/j.ultsonch.2023.106503

**Published:** 2023-06-28

**Authors:** Cuihua An, Tianyu Wang, Shikang Wang, Xiaodong Chen, Xiaopeng Han, Shuai Wu, Qibo Deng, Libin Zhao, Ning Hu

**Affiliations:** a Key Laboratory of Hebei Province on Scale-span Intelligent Equipment Technology and School of Mechanical Engineering, Hebei University of Technology, Tianjin 300401, China; b Guangdong Provincial Key Laboratory of Electronic Functional Materials and Devices, Huizhou University, Huizhou 516001, Guangdong, China; c School of Materials Science and Engineering, Tianjin University, Tianjin 300350, China; d Advanced Equipment Research Institute Co., Ltd. of HEBUT, Tianjin 300401, China

**Keywords:** Ultrasound, Cavitation effect, 2D materials, Electrocatalysts

## Abstract

Developing green, environmental, sustainable new energy sources is an important problem to be solved in the world. Among the new energy technologies, water splitting system, fuel cell technology and metal-air battery technology are the main energy production and conversion methods, which involve three main electrocatalytic reactions, hydrogen evolution reaction (HER), oxygen evolution reaction (OER), and oxygen reduction reaction (ORR). The efficiency of the electrocatalytic reaction and the power consumption are very dependent on the activity of the electrocatalysts. Among various electrocatalysts, the two-dimensional (2D) materials have received widespread attention due to multiple advantages, such as their easy availability and low price. What’ important is that they have adjustable physical and chemical properties. It is possible to develop them as electrocatalysts to replace the noble metals. Therefore, the design of two-dimensional electrocatalysts is a focus in the research area. Some recent advances in ultrasound-assisted preparation of two-dimensional (2D) materials have been overviewed according to the kind of materials in this review. Firstly, the effect of the ultrasonic cavitation and its applications in the synthesis of inorganic materials are introduced. The ultrasonic-assisted synthesis of representative 2D materials for example transition metal dichalcogenides (TMDs), graphene, layered double metal hydroxide (LDH), and MXene, and their catalytic properties as electrocatalysts are discussed in detail. For example, the CoMoS_4_ electrocatalysts have been synthesized through a facile ultrasound-assisted hydrothermal method. The obatined HER and OER overpotential of CoMoS_4_ electrode is 141 and 250 mV, respectively. This review points out some problems that need to be solved urgently at present, and provides some ideas for designing and constructing two-dimensional materials with better electrocatalytic performance.

## Highlights

• The development status of ultrasonic wave and cavitation effect are introduced.
• Various inorganic materials prepared under the assistance of ultrasound are summarized.
• The ultrasonic-assisted preparation of two-dimensional materials are discussed.
• The effects of the ultrasonic wave on the electrocatalytic performances of 2D electrocatalysts are illustrated.

## 1 Introduction

In order to tackle the current energy dilemma and environmental contamination, developing clean and efficient sustainable energy technologies has become a major research focus [1], [2], [3], [4]. The development of the advanced energy conversion technologies, such as water decomposition systems, fuel cell systems, and metal-air battery systems, is an important part of the sustainable development. Since hydrogen, oxygen, and water are readily available in nature and are environmentally friendly, electrochemical reaction systems are built around these substances in energy conversion systems. Electrochemical oxygen reduction (ORR) [5], [6], [7], oxygen evolution (OER) [8], [9], [10], hydrogen evolution (HER) [11], [12], [13], nitrogen reduction (NRR) [14], [15], and carbon dioxide reduction (CO_2_RR) [16], [17] are the core components of these new energy technologies. For example, HER and OER are the cathode and anode reactions of the water splitting. ORR is the cathode reaction of the fuel cell. However, these electrochemical reactions often require amount of precious metals or their oxides as electrocatalysts to facilitate energy conversion or fuel production, limiting their widespread application in new energy technologies. Therefore, developing cheap metal electrocatalysts with abundant reserves and equivalent or higher activity to replace them has become especially important. In the past, researchers have committed significant effort to studying the cheap metal electrocatalysts. Nevertheless, the activity of most cheap metal electrocatalysts remains low compared to that of the precious metal Pt [18], [19], [20], [21], [22], [23].

The 2D materials have attracted great attentions in all kinds of areas from the first finding of graphene [24], [25], [26]. Continuous exploration of these materials has promoted their application as electrocatalysts. In addition to graphene, a number of 2D materials have been employed to electrocatalytic reaction [27], [28], [29], [30], [31]. 2D nanomaterials have unique advantages as electrocatalysts contrasted with other structural materials: fully exposed edge sites and tunable electrochemical properties. Therefore, nitrogen-doped graphene electrocatalysts applied in zinc-air batteries, and their catalytical properties are already identical to those of traditional electrocatalysts [32], [33]. Moreover, nitrogen-doped graphene is cheap and easy to prepare on a large scale. However, the high-quality graphene is hard to be obtained under the existing technology. Another 2D material, MoS_2_, is considered the most prospective HER catalyst because S sites on the material edge have a very low adsorption energy for hydrogen protons, even near to the best Pt electrocatalyst [34], [35], [36]. Nevertheless, it is still a challenge to precisely control the active sites of the transition metal sulfides. The low cost and good performances of NiFe LDH electrocatalyst have attracted much attention, but their poor conductivity limits their wide applications. Similarly, MXene has the advantages of large specific surface area, adjustable band gap, good conductivity and abundant chelating sites. However, MXene is easily rusty in wet environment, which affects their electrical conductivity. In addition to good catalytic performance, 2D nanomaterials also have good mechanical performances and a high surface area-to-volume ratio, making them desired electrocatalysts with large-scale use.

Nevertheless, the electrocatalytic activities of most pure 2D nanomaterials are still low. For example, pure graphene has almost no activity for HER. Because the electronic structure of the electrocatalytic site at the heterophase interface is unsuitable, which weakens the adsorption strength of the reaction intermediate. Therefore, scientists have exploited various methods to ameliorate the electro-catalytical activity of graphene and other 2D nanomaterials, such as doping, defects, interfaces, alloys, additives, heterojunctions, stress, and nanostructures, which are used to regulate the electron structure of 2D nanomaterials and thus improve their electrocatalytic activities [37], [38], [39], [40], [41], [42]. These strategies also provide significant room for potential improvement in the electrocatalytic activities of 2D nanomaterials.

In recent years, various chemical synthesis methods have made great strides in development. Among them, ultrasound-assisted preparation, as a promising material synthesis tactics with the features of green, innovation, and low cost, is becoming popular. Ultrasound waves are widely used in creasming [43], foodstuff ptocessing [44], extraction [45], and other areas. The import of ultrasound can offer additional energies in the mixing, drying, and extraction processes, which has better mass transfer than those solutions that are not ultrasonically treated. In addition, various materials have been successfully synthesized via ultrasound-assisted methods, such as nanostructured materials [46], photocatalysts [47], and conductive polymers [48]. The self-assembled Ni nanocrystals have been synthesized through a simple ultrasound-assisted chemical reduction method, exhibiting good HER activity [49]. Jun et al. dispersed MXene by using 28 kHz and 580 kHz ultrasonic frequencies to obtain a new type of nanostructured material Ti_3_C_2_T_x_ MXene materials [50]. The Co_4_S_3_/Co_9_S_8_ nanosheets with mixed crystal phases have been successfully prepared for the first time by using a solvent-thermal method with ultrasonic stripping technology. Fe-Co_4_S_3_/Co_9_S_8_ nanosheets were formed after doping Fe^3+^ ions by a one-step impregnation method, which showed excellent OER activity [51].

As we all know, the constitute and the micro-structure of the 2D nanomaterials have a direct influence on the performances of the electrocatalytic reactions. Hence, the design and preparation of the 2D nanomaterials are essential. Though many kinds of 2D nanomaterials have been synthesized and applied in the electrocatalytic reactions, there are still various challenges to be addressed, such as the regulation of the surface electronic structure, the precise electrocatalytic active sites, the control of the lattice distortion and so on. The main content of this review is following. We first introduce the importance of developing 2D nanomaterials for electrocatalytic reactions and the research results of ultrasound-assisted synthesis of 2D materials. Ultrasound cavitation effect and its adhibition in the preparation of inorganic materials have been overviewed. The micro-structure, performances, advantages, and existing problems of the samples obtained by the above routes have been depicted. In the third part, the representative 2D nanomaterials for example TMDs, LDHs, graphene, MXenes, and their catalytic performance as electrocatalysts have been detailly introduced. Finally, we point out some problems of 2D nanomaterials as electrocatalysts and make prospects for their development.

## 2 Ultrasound

### 2.1 Ultrasonic wave and cavitation

The frequency of ultrasonic waves is above 20 kHz, and their applications are widespread. Ultrasonic waves can detect relevant information in a propagating medium when their intensity is low. This is called monitoring ultrasonics, for example, in ultrasonic inspection of casting shrinkage and porosity. When the intensity of ultrasonic waves is high, they can act as energy to impact a substance, known as power ultrasonics. This review only refers to power ultrasonic waves.

As the ultrasonic waves are introduced into liquid, it causes periodic pressure fluctuations in the liquid. If the amplitude of the alternating sound pressure exceeds a certain value (cavitation threshold), the nuclei of cavitation forms, grows, and ultimately cracks within the liquid or at the liquid–solid interface. Studies have shown that the crack of cavitation bubbles generates elevated temperatures, pressures, microjets [52], [53], [54], [55], [56], [57]. In a review article on acoustic cavitation published in 1980, Neppiras used the term “ultrasonic cavitation” as an academic term.

The study of cavitation can be traced back to the phenomenon of blade damage caused by cavitation on the propeller of a British destroyer in 1902. After years of research, Rayleigh published an article in 1917 titled “The Pressure Developed in a Liquid during the Collapse of a Spherical Cavity”. In the above article, he established equations that described the motion of free bubbles in incompressible fluids and indicated that the collapse of bubbles in high-speed water flow would result in extremely high pressures. Despite a century of research, the mechanism behind cavitation remains to be explored. The study of cavitation mainly focuses on two aspects: the formation of bubbles and the problem of bubble collapse based on this formation.

The formation of voids indicates the stretching of the liquid, making it a function of the liquid's tensile strength. The theoretical calculation for water's tensile strength is around 170–320 MPa. However, even in pristine water without impurities, its tensile strength only reaches 30 MPa during experiments. Scientists have proposed several hypotheses to explain this physical phenomenon, with the most widely accepted explanation being the crevice model introduced by Harvey in 1947 [58]. This theory claims that voids formed in the concave crevices of hydrophobic solid surfaces are stable in water due to their negative surface tension, making them a stable cavitation nucleus (similar to the heterogeneous nucleation theory of metal crystallization). When pressure exceeds the cavitation threshold, the voids in the crevices begin to grow.

Greenspan et al. filtered out particles (>0.2 μm) in the water, and the tensile strength of the water increased to nearly 20 MPa, which indicated that the particles in the water played a role in stabilizing nucleation [59]. The different types of particles have been added into water to study the effect of particles on cavitation [60]. The research results show that polyamide and polystyrene have significantly higher cavitation strength than other particles, because they aren’t wet with water and the surface is rough. The non-reproducibility of heterogeneous nucleation on solid particle surfaces renders the survey and study of cavitation quite tough. To investigate the effect of pits on cavitation formation, Bremond et al. used etched pits on silicon wafers [61], [62]. The experimental study proves that pit-induced effects can make cavitation occur repeatedly and controllably.

The second aspect of cavitation effect concerns the exploration of the collapse of cavitation bubbles. Rayleigh first reported that the collapse of bubbles is spherically symmetrical and generates a tremendous shock wave that causes plastic deformation of the material, leading to damage in blades. In 1944, the theory of micro-jets had been proposed, which stated that the collapse of bubbles could be non-symmetrical (flattened or diamond-shaped) under pressure gradients or near boundaries. The moment before collapse produces high-speed micro-jets that strike the wall and damage the material.

It has been observed that the pressure produced by collapsing cavitation bubbles is directly proportional to the ratio of the minimum and maximum radius of the bubbles at the moment of collapse. That is to say the smaller the minimum radius and the larger the maximum radius at collapse, the greater the generated pressure. Scholars worldwide have measured the pressure generated by the crack of cavitation bubbles using different methods. For instance, the peak pressure generated by the crack of of bubbles in degassed water have been measured under ultrasound frequency of 23.5 kHz using a piezoelectric quartz detector, which is approximately 400 MPa [63]. The peak pressure of the pulse initially increased rapidly with the amplitude of the tool rod's end face before slowing down. Using high-speed photography, Brujan et al. studied the crack of bubbles and suggested that the highest velocity of micro-jets produced at collapse can reach 500 m s^−1^, causing a water hammer pressure of 1000 MPa [64]. The mechanical mechanisms of cavitation involve the shock wave and micro-jet effects. Whether cavitation-induced damage occurs depends on whether the maximum pressure produced exceeds the material's strength and whether the collapse happens near its boundary. This is because both shock waves and micro-jets have limited effective ranges. A vast amount of theoretical and experimental studies indicate that the cavitation bubbles can damage to the material surface it only when they are at a certain distance [65], [66], [67].

**Fig. 1:**
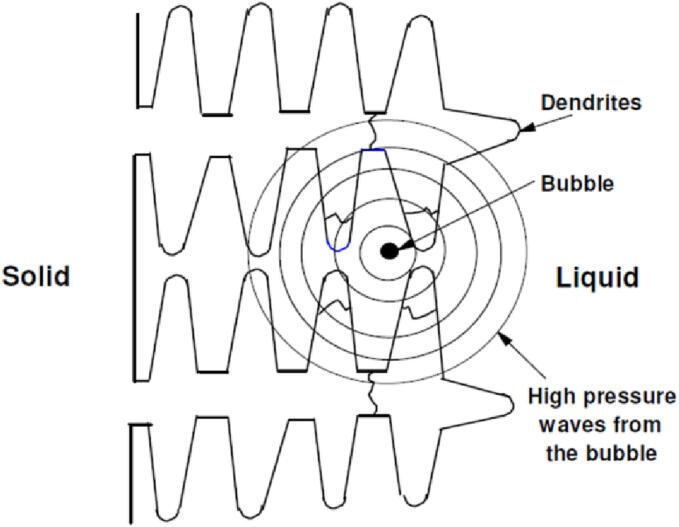
Schematic of high pressure waves generated in a solidifying melt due to violent collapse of an entrapped bubble. Reproduced with permission from [69].

Further research is needed to fully understand the ultrasonic cavitation phenomena in aluminum melts due to their high temperatures and invisibility. Recently, the synchrotron X-ray technology is attempted to study the cavitation phenomena of Al-Cu melts [68]. The generation of bubbles at the end face of the tool bar has been observed. The use of a higher acoustic power of 1500 W cm^−2^ has resulted in larger bubble diameters compared to those measured in water (120 W cm^−2^). Experimental results have shown that ultrasonic cavitation can ameliorate the grain structure of Al alloys. The potential and mechanism of ultrasonic cavitation for grain refinement in Al-Cu alloys have been studied from a theoretical perspective. Fig. 1 illustrates the concept of shock waves formed via the crack of bubbles close to dendrite crystals during solidification in Al-Cu alloys [69]. The theoretical simulation has indicated that the pressure produced by the cavitation bubble collapse decreases with the increased distance from the bubble. The maximum pressure was calculated to be 235 MPa near the bubble, decreasing to 2.5 MPa at a distance of 200 µm. However, even at this distance, the pressure still exceeded the strength of the dendrite crystal of 1.037 MPa, leading to crystal fragmentation and grain refinement during solidification.

The unique cavitation effect generated when materials workers use ultrasound has resulted in crucial achievements in the application of ultrasound in new materials preparation.

### 2.2 The adhibition of ultrasound in preparing inorganic materials

The introduction of ultrasonic treatment into chemical reactions has accelerated or controlled chemical reactions to prepare micro- or nano- powders, which has become an independent subject-sonochemistry. Suslick’s research group has carried out many creative works in sonochemistry [70], [71], [72]. This section mainly introduces the preparation of powder materials by ultrasonic pulverization. The use of ultrasonic cavitation has help to reduce the size of solid particles in liquids. Ultrasound is used to crush cells and is widely used in laboratories. In addition, ultrasound is also used to crush minerals [73], [74], [75], such as, kaolin, graphite, mica, coal powder, etc., metal Zn particles, carbon nanotubes, alumina and other ceramics, explosive materials, etc.

**Fig. 2:**
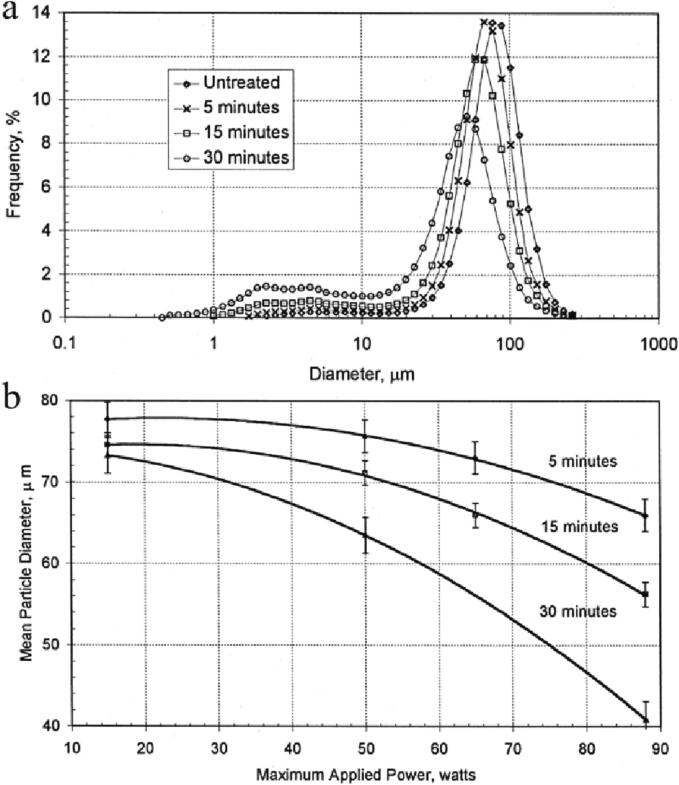
(a) The relationship between the size distribution of Al particles and the sonication time. (b) The relationship between the average particle diameter and the applied power and sonication time. Reproduced with permission from [76]. Copyright © 2000, Elsevier.

The influences of 20 kHz ultrasound power, processing time, and particle content on the average diameter and size distribution of Al particles have been studied [76]. Fig. 2a presents the change of the size distribution curve of alumina particles with increasing ultrasonic action time. The particle size distribution curve is observed to shift to the left, and the size of particles obviously reduced as the action time of the ultrasonic wave increased. In Fig. 2b, it is illustrated that the average particle size continuously decreased with the extension of processing time and the increasement of ultrasonic power. The rise in particle content resulted in an increase in particle fragmentation, indicating that the increased content of particles would not affect the occurrence of cavitation. In short, the mean diameters of the Al particles reduced with the increasement of the ultrasonic action time under the same ultrasonic power. And the average particle sizes decreased with increasing the ultrasonic power at the same ultrasonic action time.

Building on Kass's research, the grinding effect of different ultrasound delivery approaches (20 kHz tool rod delivery and 192 kHz ultrasonic cleaning equipment) on aluminum oxide particles (74–80 μm average particle size) has been explored [77]. The results show that low-frequency tool rod delivery, high power, and longer ultrasonic processing can achieve better grinding results. Due to the agglomeration of the generated nano-aluminum oxide particles, the particle size could not be detected. However, TEM results have revealed the presence of aluminum oxide particles are smaller than 100 nm.

The effects of ultrasound on the comminution of explosives and crystal particles such as NTO, AN, NaCl, HNS, HMX and RDX in heptane liquid and water have been investigated [78]. The consequence has demonstrated that the size of AN and NaCl particles didn’t change significantly, while other particles reduced from 140 μm to 20 μm after 40 min of ultrasonic treatment. At a particle size of about 40 μm, the ultrasonic input energy increased exponentially. Teipel's study on the crystal structure, surface condition and morphology of these particles have indicated that NaCl has a smooth cubic structure, making it less susceptible to ultrasonic comminution [78]. HNS is needle-shaped and requires only a small energy input to be comminuted in its longitudinal direction. HMX and RDX have irregular surface morphology, making them more susceptible to comminution.

**Fig. 3:**
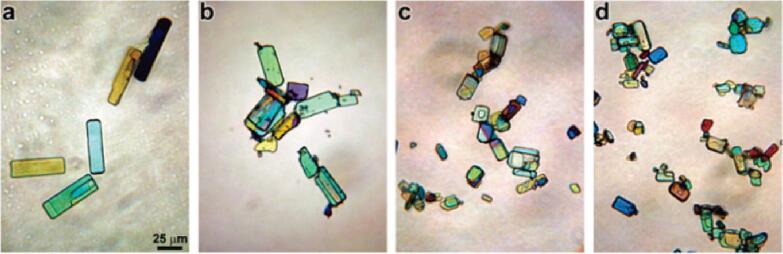
The optical micrographs depicting the sonofragmentation of aspirin crystals: sonication for 0 min (a), 1 min (b), 3 min (c), 10 min (d). The power and frequency of the sonication are 10 W and 20 kHz, respectively. The scale bar of all images is 25 μm. Reproduced with permission from [79]. Copyright © 2011, American Chemical Society.

In 2004, Geim et al. first obtained single-layer graphene via a manual peeling method. The specifical procedure was following: the transparent tape was firstly used to stick to the surface of the graphite. Then the transparent tape was fold in half, sticked on and pulled off. In this way, both sides of the transparent tape were stuck with a graphite layer. And the graphite layer became thin again. Repeated this many times, when the graphite layer on the transparent tape was as thin as one carbon atom, it became graphene. Subsequently, researchers have conducted extensive creative work to prepare graphene for large-scale production. The ultrasonic exfoliation has been utilized to peel graphite layers in a liquid medium [74]. The ultrasonic energy can exfoliate the graphite layer by layer to obtain up to 12 wt% high-quality non-oxidized single-layer graphene without introducing any defects, which benefit the excellent properties of graphene. The crushing effect and mechanism of ultrasound on aspirin crystals have been studied [79]. The polarized light images of aspirin particles at different ultrasonic treatment times have been show in Fig. 3. With the increasement of the ultrasonic treatment time, the particle size reduces while the number of particles per unit area rises significantly. Since the size of the particle is less than 200 μm, the bubbles don’t rupture asymmetrically, and the shock waves formed via cavitation crack are the dominating mechanism for crushing aspirin particles. The above results have indicated that the pulverization effects of ultrasound on particles are influenced by various parameters, including particle size and shape, particle strength, properties of liquid media, ultrasonic action mode and intensity, processing time, liquid volume, particle content and so on.

Ultrasonic cavitation is mainly used for degassing of metals and refinement of solidified structures in the early stage. With the progress of high-power ultrasonic facility and the embedded research of the acting regime of the ultrasonic wave, the cavitation effect produced by strong ultrasonic waves in liquids is gradually applied to improve wettability and disperse micro-particles and even nanoparticles. We will concentrate on the ultrasonic-assisted preparation of 2D materials and their application in electrocatalytic reactions.

## 3 Ultrasound-assisted synthesis of 2D nanomaterial electrocatalysts

2D nanomaterials, especially TMDs and graphene, have garnered broad attention due to the following numerous advantages. Notably, they are easily accessible and cost-effective, and possess tunable physicochemical properties that enable their development as replacements for precious metal electrocatalysts [80], [81], [82], [83]. For instance, researchers have found that nitrogen-doped graphene exhibits greatly enhanced ORR performance in alkaline condition compared to pure graphene, which is close to the commercial 20 wt%Pt/C [84]. Additionally, MoS_2_ demonstrates high HER activity in acidic condition [85]. Despite some progress, the electrocatalytic activity and stability of most 2D materials still require improvement. Currently, apart from Pt, the majority of materials exhibit inferior acidic ORR activity. The graphene shows much lower activity in acidic ORR than alkaline ORR [86], [87]. Moreover, transition metal dichalcogenide materials continue to possess higher HER reaction energy barriers than metal Pt, leading to greater energy consumption in hydrogen production [88], [89]. The electrocatalytic reactions of 2D material occur at the active sites of heterogeneous interfaces, and the electron structure of these activated sites directly influences the size of the reaction energy barrier, thereby determining the starting potential for electrochemical reactions and the reaction rate, which ultimately affects energy consumption. The following sections will focus on four types of 2D nanomaterial catalysts: TMDs, LDHs, graphene, and MXenes.

### 3.1 Tmds

Various TMDs have also been prepared by the ultrasound-assisted method [90], [91], [92], [93], [94], [95] and studied as electrocatalysts, especially MoS_2_-based materials. DFT calculations have shown that its electronic structure is very similar to that of Pt. Additionally, similar to other transition metal phosphides, S interacts with protons, promoting the HER reaction [96]. Since the active sites at MoS_2_ edges have extremely supernal catalytic activities, the focus of research has been on preparing nanomaterials with high edge site densities and defect formations in their structures [97].

**Fig. 4:**
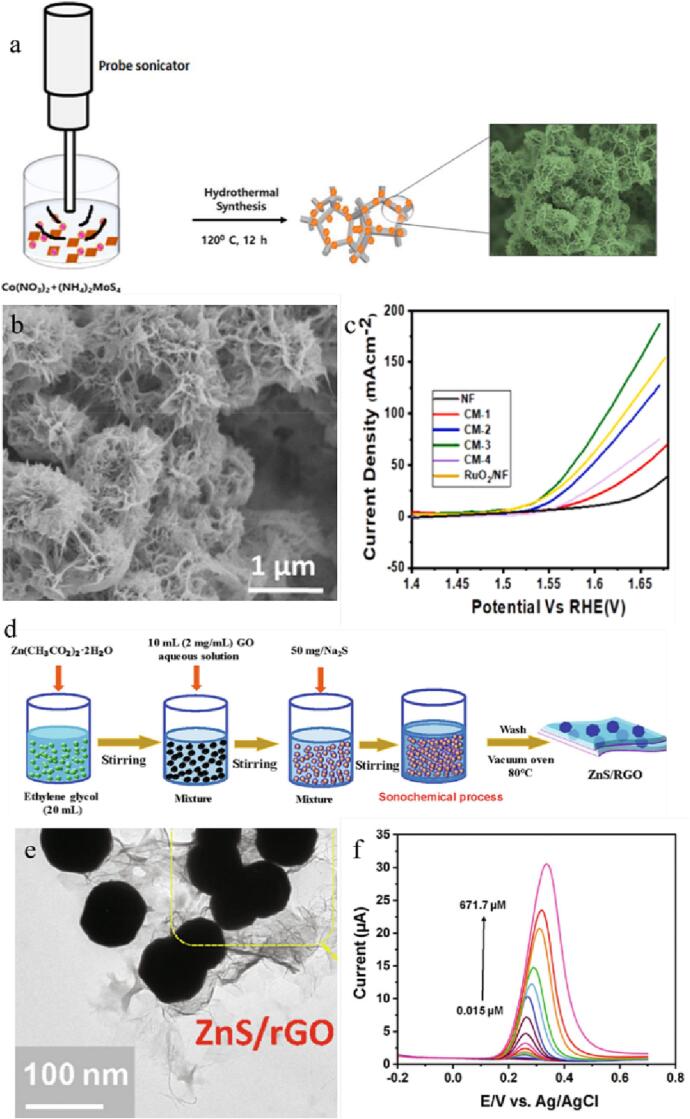
(a) Scheme for the preparation of porous 3D CoMoS_4_ electrocatalyst. (b) SEM image. (c) LSV curves. Reproduced with permission from [98]. Copyright © 2021, Elsevier. (d) Scheme for the synthesis of ZnS NPs/RGO nanocomposite via sonochemical method. (e) TEM image of ZnS NPs/RGO nanocomposite. (f) Differential pulse voltammetry curves. Reproduced with permission from [99]. Copyright © 2020, Elsevier.

The three-dimensional (3D) nano-flower-like porous CoMoS_4_ electrocatalysts have been synthesized through a facile ultrasound-assisted hydrothermal method (as shown in Fig. 4a) [98]. Because of the luxuriant active places, small electrolyte contact resistance, and rapid electron transition rate on CoMoS_4_ electrocatalysts (Fig. 4b), they exhibit excellent electrocatalytic activity. When CoMoS_4_ is used as the cathode and anode in electrolysis, a 10 mA cm^−2^ current density is achieved at 1.51 V. Additionally, the OER overpotential of CoMoS_4_ electrode is 250 mV (Fig. 4c) and the HER overpotential is 141 mV. Furthermore, the vesicular structure of CoMoS_4_ material offers a perfect pathway for the spread of the electrolyte, improves its reaction dynamics, and promotes gas emission.

A composite material of ZnS nanoparticles (ZnS NPs) coated with RGO has been synthesized using ultrasonic bath [99]. The preparation process is shown in Fig. 4d, where the reaction was carried out in an ultrasonic bath for 1 h (50 kHz and 60 W), followed by calcination to obtain ZnS NPs@RGO nanocomposites. Because of the strong synergistic action between RGO and ZnS NPs, the electrode material modified with ZnS NPs@RGO exhibits high stability and excellent sensitivity, making it suitable for determining 3,4-dihydroxycinnamic acid in different foods (Fig. 4e-f).

**Fig. 5:**
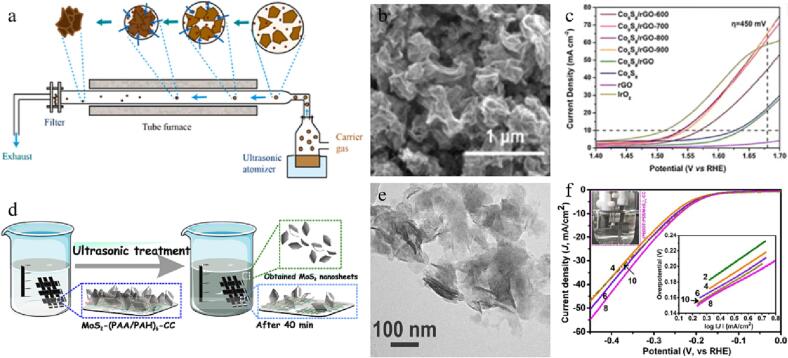
(a) Scheme of the ultrasonic spray pyrolysis system. (b) SEM image of Co_9_S_8_/RGO after heat treatment at 700 °C. (c) LSV curves of various Co_9_S_8_/RGO. Reproduced with permission from [100]. Copyright © 2022, Elsevier. (d) Scheme for the process of stripping the MoS_2_ nanosheets off the carbon cloth base through the ultrasonic treatment. (e) TEM image of MoS_2_ nanosheets. (f) LSV curves. The relevant Tafel slope is the inset. Reproduced with permission from [101]. Copyright © 2021, Springer.

Yang et al. exploited a new route for constructing a three-dimensional rGO/Co_9_S_8_ composites with extraodinary synergetic actions between the two components [100]. This was achieved by employing a simple process that combined ultrasound atomization drying and thermal treatment (Fig. 5a). The resulting Co_9_S_8_/rGO composite material exhibits an excellent in-plane shrinking structure, leading to a large specific surface area and abundant potential electrocatalytic places (Fig. 5b). The optimized electrocatalyst shows 0.308 V overpotential and of 130.0 mV dec^-1^ Tafel slope at 10 mA cm^−2^, and also demonstrates excellent cycling consistency in 1 M KOH (Fig. 5c). The above work offers a potential large-scale tactic for the preparation of TMDs oxygen evolution reaction electrocatalysts, which will contribute to the progress of hydrogen energy study.

Ultrathin structures of transition metal dichalcogenides possess various superior versatile performances and has huge potential in energy conversion fields. However, their simple preparation and volume production are still tough. MoS_2_ nanoflakes with 100 nm lateral size and 10–20 nm thickness have been prepared using inorganic matrix-facilitated deposition and ultrasonic exfoliation techniques (Fig. 5d) [101]. They exhibit representative various absorptions, suggesting the particular band structure features of MoS_2_ 2D nanosheets (Fig. 5e). In addition, the electron structure of slim MoS_2_ nanosheets can be regulated by tunning the surrounding environment in which they are assembled. In the interlayer interfacial assembly of (PEDOT:PSS/MoS_2_)_n_ multilayer films, the charge transition procedure enhances the catalytic efficiency of MoS_2_ nanosheets. The catalytic performance of the assembly is optimized through a well-regulated layer-by-layer procedure, and the assembly exhibits efficient and stabilized HER performances (Fig. 5f). We anticipate the above preparation tactic of MoS_2_ 2D nanosheets can facilitate the study of versatile materials, which can be generalized to synthesizing other nanosheets.

### 3.2 LDHs

LDHs is a kind of ionic layered compounds composed of solvent molecules, interlayer anions, and positively charged main layer [102]. The bivalent positive charges are partially replaced by trivalent cations coordinated by hydroxyl octahedrons, and the supererogatory cations are balanced by interlayer negative ions. The common symbol of LDHs is [M^2+^_1-x_M^3+^_x_(OH)_2_]^z+^A^n-^_z/n_·mH_2_O, in which M^2+^ and M^3+^ are + 2 and + 3 metal cations, and A^n-^ is interlayer negative ion [103]. Every hydroxyl radical in the LDH layer towards the interlayer area, which may generate hydrogen bonds with water molecules or interlayer negative ions. Various LDHs have been synthesized via the ultrasound-assisted method [104], [105], [106], [107], [108], [109], [110], [111], [112]. Various metals can be imported into LDHs, and the distances and negative ions between layers can be regulated by the exchange of the negative ions. Hence, the constitute of the LDHs can be adjusted at will, which further widen the applications of the LDHs [113], [114]. In addition, the stripping of the liquid can expand the distance between layers and weaken the interaction between motherboards, thus forming ultra-thin nanosheets with coordinated unsaturated active sites and improving the OER performances. Ni/Fe-LDH is considered to be an effective OER catalyst in alkaline solution.

**Fig. 6:**
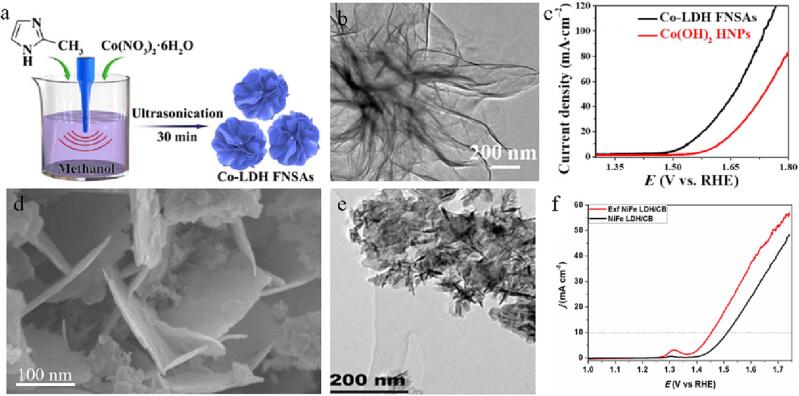
Scheme of the preparation route (a) and TEM image (b) of Co-LDH FNSAs. (c) LSV curves of Co-LDH FNSAs and Co(OH)_2_ HNPs. Reproduced with permission from [112]. Copyright © 2020, Springer. SEM (d) and TEM (e) images of Exf NiFe LDH/CB. (f) LSV curves of NiFe LDH/CB and Exf NiFe LDH/CB. Reproduced with permission from [116]. Copyright © 2019, Elsevier.

Chen et al exploited a facile ultrasonic synthesis route, which can produce flower-like cobalt base-like dihydroxide nanowires on a large scale within 30 min (Fig. 6a-b) [115]. The nanoflower has excellent electrocatalytic performance and excellent stability under alkaline conditions. The overpotential, the Tafel slope and the charge transfer resistance are 300 mV, 110 mV·dec^-1^ and 21.4 Ω at 10 mA·cm^−2^, which is superior to the commercialized RuO_2_ nanoparticles (Fig. 6c). This is because of the affluent active places and redox coupling brought by the 2D ultra-thin structure of cobalt-like base-like dihydrooxides nano-sheet aggregates, which further improves the OER activities.

**Fig. 7:**
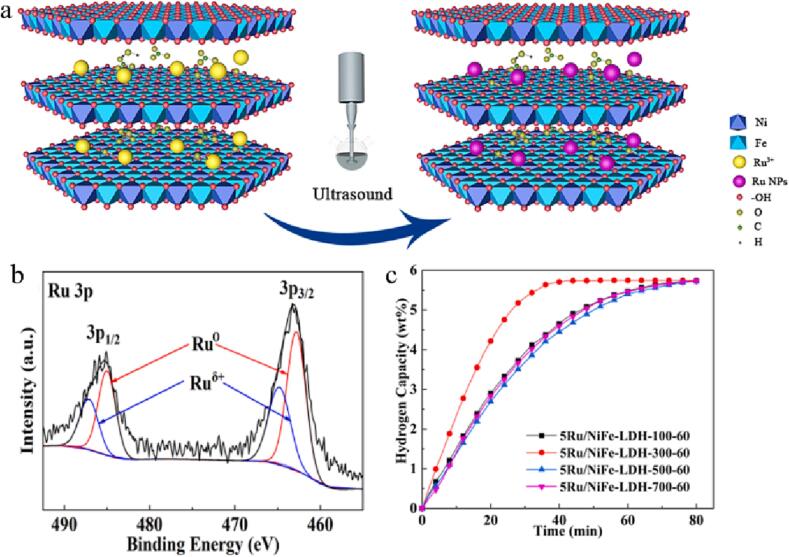
(a) Mechanism illustration of Ru/NiFe-LDH synthesized via ultrasound-assisted preparation means. (b) XPS spectrum for Ru 3p. (c) Hydrogenation plots of various Ru/NiFe-LDH catalysts synthesized at different ultrasonic power. Reproduced with permission from [118]. Copyright © 2021, Elsevier.

NiFeLDH/ carbon black (CB) bulk materials have been stripped into monolayer or small-layer NiFeLDH/CB nanoflakes via a simple ultrasonic peeling method (Fig. 6d-e) [116]. The ultrasonic stripped NiFeLDH/CB nanowires in pure water have excellent OER activity, which is better than other cheap metal catalysts. In alkaline solution, the overpotential of the exfoliated NiFeLDH/CB nanowires is 220 mV (Fig. 6f) and 35 mV dec^-1^ Tafel slope, which is superior to the bulk NiFeLDH/CB (280 mV overpotential and 48 mV dec^-1^ Tafel slope at 10 mA cm^−2^). Meanwhile, after 12 h, the structure of the stripped NiFeLDH/CB remains stable and the potential is nearly constant, suggesting its high stability. Similarly, Li et al. used ultrasound-assisted method to insert formamide into the interlayer of NiFeLDH materials to expand the interlayer spacing, which increased the active places and provided greater space for the spread of reactants, bubbles [117]. The results show that after formamide is inserted at 80 ℃, the distance of NiFeLDH interlayer enlarged from 7.8 to 9.5, which improve the OER activities (210 mV overpotential), interfacial connectivity and stability. Highly active Ru nanoparticles (RuNPs) have been loaded on NiFe LDH (Ru/NiFe-LDH) by ultrasound-assisted route with no reducing and stabilizing agents (Fig. 7a) [118]. Ultrasound excites the rich hydroxyl radicals on the NiFe-LDH surface to form hydrogen free groups (∙H), reducing Ru from 3 valence to zero valence (Fig. 7b). In Fig. 7c, the N-ethylcarbazole (NEC) hydrogenation rate firstly increased and then decreased with the rise of ultrasonic power. When the ultrasonic power was 300 W, the hydrogenation rate was the fastest. And after the NEC hydrogenation reaction of 40 min, the mass hydrogen storage capacity remained unchanged. Oxygen-containing groups enhance the interaction between layered NiFe-LDH carriers and Ru nanoparticles, thus making the structure of Ru/NiFe-LDH more stable and ameliorating the electrocatalytic activities of hydrogenation (Fig. 7c).

The petal-shaped NiCoFe layered double hydroxides (LDHs) have been prepared after 60 min of high-power ultrasound [119]. The morphology of nano-petal-like NiCoFe_50_LDH will be affected by ultrasonic time, and 60 min is the best time to synthesize NiCoFe-LDH. The above consequences manifest that compared with NiCoFe-LDH nanoparticles (synthesized in 30, 120 mins by coprecipitation and ultrasonic method), the nano-flake NiCoFe-LDH has better OER properties (0.46 V starting voltage, 227 mV overpotential and 234 mV·dec^-1^Tafel slope at10 mA·cm^−2^) and stable cycling performances in 0.1 M KOH. The above means of synthesizing nanometer petal NiCoFe LDH is simple and easy to operate, which can be extended to large-scale industrial production.

The ultrafine ZnCo-LDH nanowires (ZnCo-UF) with 3.5 nm average particle size and 0.5 nm thickness have been successfully exfoliated from the massive ZnCo materials by simple ultrasonic peeling method [120]. The monolayer ZnCo-UF nanowires are rich in oxygen vacancies and unsaturated coordination, which endow them with semi-metallic properties, enhance the charge transfer, improve the positivity of the nanowires, and enhance the adsorption capacity and OER activities of the intermediates. The OER activities of these ultra-fine ZnCo-UF nanowires (overpotential 340 mV@5 mA·cm^−2^) are dramatically superior to bulk materials (overpotential 530 mV@5 mA·cm^−2^). This synthesis method is universal and has been successfully applied to NiFe-LDH, CoMn-LDH and CoFeAl-LDH materials.

### 3.3 Graphene

Non-metallic materials are composed of metal-free atoms, such as various carbon materials, which are widely used in ORR, OER and HER [121], [122], [123]. At present, scientists have put a great deal of energy to develop non-metallic materials into catalysts to replace Pt and Ru/Ir-based materials. Generally speaking, 2D graphene nanosheets have large specific surface area and thinner thickness, layered structure, porous structure, excellent physical, chemical properties, which render them the most competitive research objects in the next generation of electrochemical energy devices. A mass of literatures have accounted for the successful synthesis of 2D graphene nanosheets with specific physical chemistry properties via ultrasound-assisted method [124], [125], [126].

**Fig. 8:**
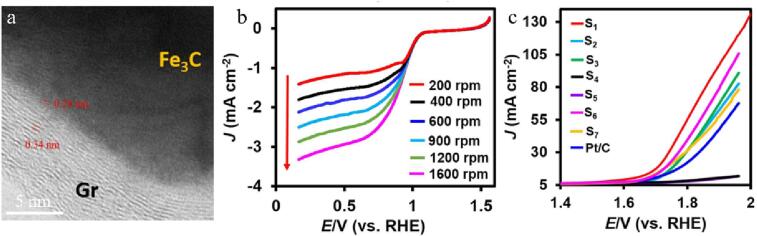
(a) TEM image of NS-GR/Fe_3_C. (b) LSV plots of NS-GR/Fe_3_C modified electrode (RDE) at various rotation rates. (c) LSV curves of various NS-GR/Fe_3_C electrocatalysts and commercial Pt/C. Reproduced with permission from [128]. Copyright © 2020, Elsevier.

The Pt and Sn precursors, graphene oxide (GO) have been treated by the ultrasonic wave with 20 kHz frequency. The Pt/Sn/rGO catalysts have been prepared by loading the monometallic Pt or the bimetallic Pt-Sn nanoparticles on the surface of rGO [127]. This sonochemical method is a simple method to obtain monoatomic and diatomic nanoparticles with different compositions, which can distribute these metal nanoparticles more evenly on rGO. Graphene as the matrix can better fix metal particles, increase the active places on the surface of the electrode, and significantly improve the catalytic efficiency of metal nanoparticles. The nitrogen-sulfur co-doped graphene/Fe_3_C (NS-GR/Fe_3_C) nanocomposites have been prepared by the assist of the ultrasound with homogenization dispersion advantage (Fig. 8a) [128]. The results show that the NS-GR/Fe_3_C catalyst prepared under the ultrasonic bath of 150 W and 40 kHz has outstanding performance for both ORR and OER processes (Fig. 8b-c). Due to the influence of heteroatom doping, Fe_3_C has a strong synergistic effect with graphene sheets. The doped matrix has high electrical conductivity and electrocatalytic activity, which makes the NS-GR/Fe_3_C catalyst show excellent catalytic performance in aqueous and non-aqueous electrolytes.

**Fig. 9:**
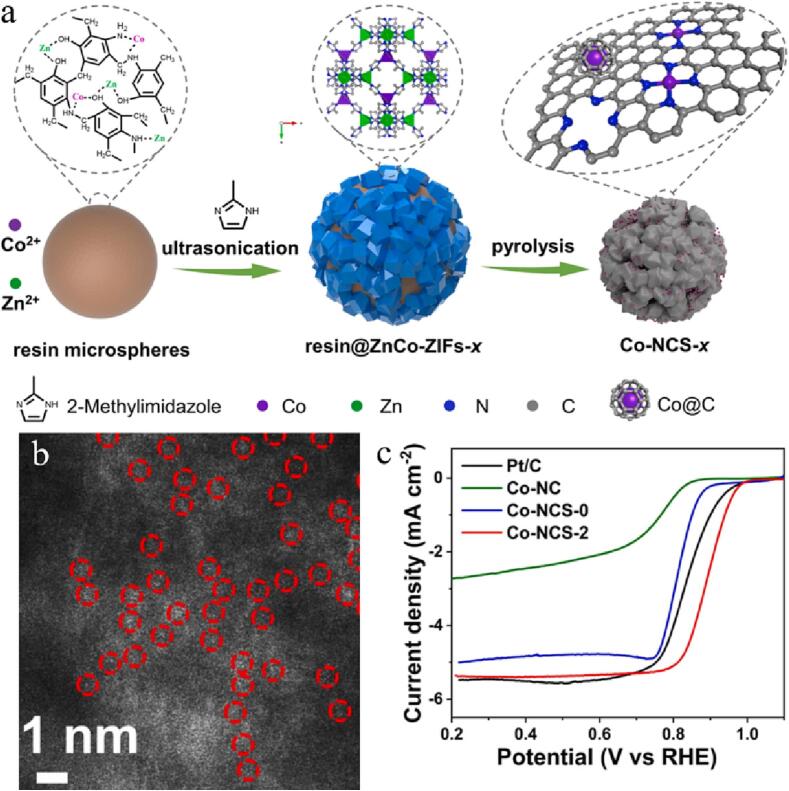
(a) Schematic illustration of the synthesis of Co-NCS-x. (b) HAADF-STEM image of Co-NCS-2. (c) LSV curves of various Co-NCS electrocatalysts and commercialized Pt/C. Reproduced with permission from [129]. Copyright © 2021, Elsevier.

Mai et al reported that layered N-doped carbon spheres derived from bimetallic zeolite imidazole skeleton (BMZIF) were grappled by graphene-coated cobalt nanocrystals (Co@C) and cobalt monoatom (CoSAs) (Co-NCS) for efficient catalysis of ORR [129]. The authors use ultrasound-assisted technology to disperse BMZIF on phenolic resin microspheres and obtain Co-NCS-x after pyrolysis (Fig. 9a). The high pressure and temperature caused by the ultrasonic wave promotes nucleation and greatly shortens the synthesis time. The ultrasonic method can obtain smaller particles and more uniform distribution than traditional methods (Fig. 9b). BMZIF with interconnected porous carbon skeleton can promote electronic transmission and contains a mass of active places, so that Co-NCS-2 shows excellent ORR activities (Fig. 9c). The nickel manganese oxides modified partially rGO (NiMnO@pr-GO) nanocomposites have been synthesized by sonochemical means for the modification of glassy carbon electrode (GCE) [130]. CV outcomes show that the NiMnO@pr-GO nanocomposites present higher electro-catalytical activities and lower voltage than that of single NiMnO and GO.

Co-Sn-Pd supported on reduced graphene has been prepared through ultrasonic radiation [131]. Even at very low content of Pd, tribasic Co-Sn-Pd/rGO electro-catalysts exhibit higher ORR activity and stability than dibasic Pd-Co/rGO catalysts and 20 wt% Pt/C catalysts. 2D Pd nanowires have been loaded on GO (Pd/rGO-u) by sonochemical method [132]. By contrast with Pd/rGO-c synthesized by traditional method, Pd/rGO-u presents superior ORR electrocatalytic properties and follows 4e^-^ reaction dynamics.

### 3.4 MXene

Thanks to the high conductivity, high hydrophilicity, adjustable band structure and 2D nanostructures conducive to charge transport, MXene has attracted extensive attention in the area of electrocatalysis since its advent. Researchers have made great progress in HER, OER, ORR and so on. Compared with hydrothermal and solvothermal methods, mechanically assisted ultrasound doesn’t need the harsh conditions of high pressure and temperature. The reaction mechanism depends on the exfoliation and cracking of the layer. Among them, the mode of ultrasound (probe ultrasound, water bath ultrasound), ultrasonic power and time affect the final formation and yield of MXene. At present, a series of MXene-based composites have been synthesized by ultrasound-assisted method [133], [134], [135].

Ti_3_C_2_T_x_ MXene is usually obtained by etching the main group elements in its corresponding transition metal carbide MAX phase. Because of its particular physical and chemical performances, it shows excellent electrochemical performance in the electrocatalytic reaction. Nevertheless, the lowered efficiencies of getting rid of dominating radical elements seriously limits the adhibition prospect of MXene and MXene-based composites. In view of this, Ti_3_C_2_T_x_ MXene materials have been synthesized using Ti_3_AlC_2_ as the precursor via an ultrasound-assisted method [136]. Under the condition of ultrasound, the chemical etching efficiency of Ti_3_AlC_2_ is greatly improved, and the etching time in dilute HCl solution is shortened from 24 h to 8 h. In particular, with the assistance of ultrasound, a higher etching efficiency can be achieved in 2 % HF solution, while the 2 % concentration is lower than the currently reported etching environment. The Ti_3_C_2_T_x_ MXene material obtained by 8-hour ultrasound shows the best electrochemical performances. This ultrasound-assisted process can remove the AlF_3_ impurities formed on the etchant surface more efficiently, and the acoustic cavitation effect produced in the ultrasonic treatment process can destroy the hydrogen bond between the two adjacent layer functional groups.

**Fig. 10:**
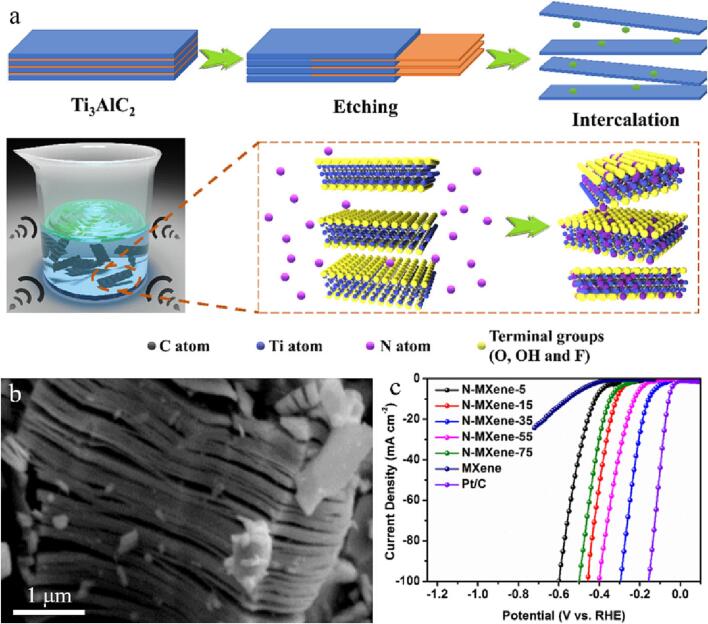
(a) Schematic illustration of synthesizing N-MXene. (b) SEM image of N-MXene-35. (c) LSV curves of various N-MXene electrocatalysts and commercial Pt/C. Reproduced with permission from [137]. Copyright © 2021, Elsevier.

**Fig. 11:**
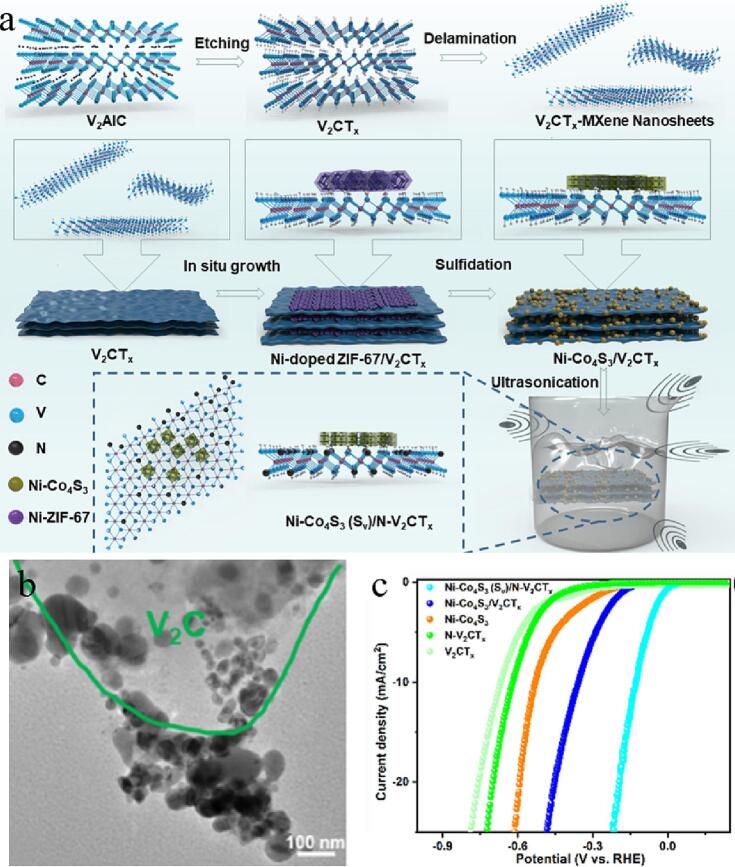
(a) Schematic illustration of the preparation of Ni-Co_4_S_3_ (S_v_)/N-V_2_CT_x_. (b) TEM image of N-MXene-35. (c) LSV curves of various catalysts. Reproduced with permission from [138]. Copyright © 2023, Royal Society of Chemistry.

Han et al exploited a facile and rapid means to continuously regulate the nitrogen doping of MXene through cryogenic ultrasound [137]. Firstly, a simple ultrasonic method with adjustable temperature is used to realize the continuous control of nitrogen concentration in MXene (Fig. 10a). Secondly, nitrogen doped into MXene not only provides an efficient electronic transition path, but also increases the spacing of MXene interlayer, thus exposing additional active places and producing excellent electrocatalytic performance (Fig. 10b). Through the generation of Ti-N bond at the optimal ultrasonic temperature, the surface of MXene can be embellished and the HER activities can be further enhanced. Third, the optimized nitrogen-doped MXene catalyst has low overpotential (162 mV), low Tafel slope and good durability at 10 mA·cm^−2^, thus providing excellent HER electrocatalytic properties (Fig. 10c).

heteroatom doping and vacancy engineering of MXene-based composites have attrested wide attention. Nandi et al used S vacancy regulated nickel-doping Co_4_S_3_ nanocubes/N-doping V_2_CT_x_ MXene nanowires (Ni-Co_4_S_3_ (S_v_)/N- V_2_CT_x_) electrocatalysts. And the vulcanized process is based on the basis of ultrasonic treatment (as shown in the Fig. 11a) [138]. The effects of sulfur vacancy, hollow structure and Ni-N doping on the electrocatalytic activity were studied systematically (Fig. 11b). The vacancies in Ni-Co_4_S_3_ (S_v_) hollow nanocubes reduces the contact resistance and promotes the electrons immitting from the surface to the active center. This particular porous construction can provide ample land areas to promote electrolyte infiltrating and effective transition of the electrons and ions. Heterodoping can availably solve the stacked issuse of MXene, expose abundant active places and accelerate electron transition. Therefore, the prepared Ni-Co_4_S_3_ (S_v_)/N-V_2_CT_x_ has excellent HER activities with 127 mV overpotential (Fig. 11c). Overall, MXene-based electrocatalysts have the remarkable HER electrocatalytic properties due to their sulfur vacancies, hollow porous structure and Ni-N doping. MXene with CoBDC nanowires have been coupled to obtain tightly connected 2D/2D heterostructures using a simple ultrasound-assisted growth means [139]. Versatile MXene with ideal hydrophilicity and high conductivities can be used as bridging carriers to guarantee structure stability and expose more active places. In addition, the Co-O-Ti bond generated at the interface availably strikes the charge transport and adjusts the electron structure of the Co active places, thus enhancing the dynamics. Therefore, the optimal CoBDC/MXene shows a low HER overpotential of 76, 41 and 29 mV in neutral, acidic and alkaline electrolytes. Theoretical calculations show that the electron redistribution caused by interface bridging optimizes the free energy of hydrolysis ionization and H* adsorption, thus improving the energy barrier of HER reaction. The above research not only offers a new type catalyst for high efficiency HER under wide pH ranges, but also develops a fire-new way to design highly catalytic activity system.

## 4 Conclusion and prospect

Under the background of carbon neutralization, the electrocatalytic reactions have attracted much attention due to the excellent performance in new energy field. The design and fabrication of the electrocatalysts are crucial to the electrocatalytic reactions. We review some latest developments in ultrasound-assisted preparation of 2D material catalysts and classified them according to different types of materials. This review focuses on the cavitation effect and its application in synthesizing inorganic materials, and deeply discusses the representative 2D materials via the ultrasound-assisted method, such as transition metal sulfides, layered bimetal hydroxides (LDH), graphene, MXene, and their catalytic properties as electrocatalysts. Finally, we draw the following conclusions: 1) The use of ultrasonic cavitation is of great significance in the preparation of inorganic materials. Although it is only an auxiliary means for synthesizing inorganic materials, practice shows that the import of ultrasonic cavitation greatly expedites the rate of synthesis and preparation of inorganic materials, and greatly improves the uniformity and dispersion of the separation. As we all know, the ultrasonic cavitation is closely related with the ultrasonic parameters. However, the relationship between ultrasonic parameters (amplitude, frequency, etc.) and the microstructure and electro-catalytical activities of the material is not clear. Therefore, it is necessary to clarify the relationship between ultrasonic parameters (ultrasonic cavitation) and the structure of catalyst materials for guaranteeing the accurate synthesis of catalyst active places, simplify the process and reduce the cost. Finally, it is necessary to implement the massive commercialized preparation of 2D materials. 2) The appropriate layer distance can enhance the electro-catalytic properties of 2D materials. But the layered structure is easy to collapse in the process of electrocatalytic cycle, resulting in the difficulty of synthesizing highly active 2D materials. The intercalation strategy can effectively alleviate the change of layered structure, so it is easier to prepare 2D materials with high stability.

According to the above description, cheap 2D material catalysts have presented outstanding performance and enormous potential in electrocatalysis area, but some problems have still to be resolved: 1) 2D transition metal sulphide materials are rich and varied. In this review, only a few representative materials are discussed, and there are many unstudied objects. And the prepared 2D molybdenum sulphide nanosheets with lattice distortion show excellent electrocatalytic activity. However, the accurate control of its surface lattice distortion has not been realized. Therefore, the micro-nano processing technologies should be developed to prepared the 2D MoS_2_ nanosheets with a particular lattice distortion. 2) Because of the presence of two metals in transition metal-based LDHs system, the selection of metal types, especially the regulation of metal surface electronic structure, directly affects the catalytic activities. However, the precise regulate of its surface electronic structure has not been achieved yet. Therefore, various binary LDHs materials should be synthesized and their surface electronic structures should be characterized, which is helpful to generate the database for guiding the preparation of the electrocatalysts with precise surface electronic structure. 3) Graphene and MXene have low intrinsic activity, which are usually as substrates. They show excellent electrocatalytic activity when compounded with other materials. But their catalytic mechanism is not clear. Therefore, through in-situ analysis technology, machine learning and other new means to further deepen the understanding of the catalyst structure-activities relationship and process mechanism under the condition of dynamic electrochemical reaction, and further guide the intellectually design of the electrocatalysts. 4) The unified standards are lacking for testing the electro-catalytical performances of the prepared 2D material catalysts, such as test conditions, the standards of the commercial Pt/C used for comparison, and parameters which can correctly reflect the performance of the catalyst. Therefore, the uniform standards should be developed by the global researchers in order to better compare the electro-catalytical performances of various electrocatalysts.

In a word, 2D materials synthesized by ultrasound-assisted method have enormous potency in electrocatalytic reaction, which demands us to uncover the distinct micro-structure-activities relationship between the micro-structures and electrocatalytic performance 2D material. Hence, scientists should take into account to prepare electrocatalysts with clear active places, and establish a corresponding model for theoretical calculation according to the characterization results, so as to provide guidance for designing 2D materials with outstanding electrocatalytic properties. It is believed that 2D materials will effectively promote the evolution of novel energy techniques in the immediate future, so as to realize the double carbon goal as soon as possible.

## CRediT authorship contribution statement

**Cuihua An:** Conceptualization, Investigation, Data curation, Writing – original draft. **Tianyu Wang:** Writing – original draft. **Shikang Wang:** Writing – original draft. **Xiaodong Chen:** Supervision, Writing – review & editing. **Xiaopeng Han:** Supervision, Writing – review & editing. **Shuai Wu:** Writing – review & editing. **Qibo Deng:** Supervision, Project administration, Writing – review & editing, Funding acquisition. **Libin Zhao:** Supervision, Project administration. **Ning Hu:** Supervision, Funding acquisition.

## Declaration of Competing Interest

The authors declare that they have no known competing financial interests or personal relationships that could have appeared to influence the work reported in this paper.

## Data Availability

No new data were created or analyzed in this study. Data sharing is not applicable to this review article.

## References

[1] Chen F.Y., Wu Z.Y., Adler Z., Wang H.T. (2021). Stability challenges of electrocatalytic oxygen evolution reaction: from mechanistic understanding to reactor design. Joule.

[2] Yu Z.Y., Duan Y., Feng X.Y., Yu X.X., Gao M.R., Yu S.H. (2021). Clean and affordable hydrogen fuel from alkaline water splitting: past, recent progress, and future prospects. Adv. Mater..

[3] Khan U., Nairan A., Gao J.K., Zhang Q.C. (2022). Current progress in 2D metal-organic frameworks for electrocatalysis. Small Struct..

[4] Wang H., Li J.M., Li K., Lin Y.P., Chen J.M., Gao L.J., Nicolosi V., Xiao X., Lee J.M. (2021). Transition metal nitrides for electrochemical energy applications. Chem. Soc. Rev..

[5] Jiao P.G., Ye D.H., Zhu C.Y., Wu S., Qin C.L., An C.H., Hu N., Deng Q.B. (2022). Non-precious transition metal single-atom catalysts for the oxygen reduction reaction: progress and prospects. Nanoscale.

[6] Liu J., Gong H., Ye G., Fei H. (2022). Graphene oxide-derived single-atom catalysts for electrochemical energy conversion. Rare Metals.

[7] Wang C., An C.H., Qin C.L., Gomaa H., Deng Q.B., Wu S., Hu N. (2022). Noble metal-based catalysts with core-shell structure for oxygen reduction reaction: progress and prospective. Nanomaterials.

[8] Li S., Gao Y., Li N., Ge L., Bu X., Feng P. (2021). Transition metal-based bimetallic MOFs and MOF-derived catalysts for electrochemical oxygen evolution reaction. Energy Environ. Sci..

[9] Zhang Y.C., Han C.D., Gao J., Pan L., Wu J.T., Zhu X.D., Zou J.J. (2021). NiCo-based electrocatalysts for the alkaline oxygen evolution reaction: a review. ACS Catal..

[10] Ren X., Li X., Peng Y., Wang G., Yin J., Zhao X., Wang W., Wang X. (2022). FeNiS2/reduced graphene oxide electrocatalysis with reconstruction to generate FeNi oxo/hydroxide as a highly-efficient water oxidation electrocatalyst. Rare Metals.

[11] Zhou B.H., Gao R.J., Zou J.J., Yang H.M. (2022). Surface design strategy of catalysts for water electrolysis. Small.

[12] Foroughi F., Bernacker C.I., Rontzsch L.G., Pollet B. (2022). tanding the effects of ultrasound (408 kHz) on the hydrogen evolution reaction (HER) and the oxygen evolution reaction (OER) on raney-Ni in alkaline media. Ultrason. Sonochem..

[13] Yang Z., Du X., Ye X., Qu X., Duan H., Xing Y., Shao L., Chen C. (2021). The free-standing nanoporous palladium for hydrogen isotope storage. J. Alloys Compd..

[14] Xu C., Huang J., Ma J. (2021). Green, cheap and rechargeable Al-N_2_ battery with efficient N_2_ fixation. Rare Metals.

[15] Li H.D., Zhao H., Li C.P., Li B.Q., Tao B.R., Gu S.A., Wang G.F., Chang H.X. (2022). Redox regulation of photocatalytic nitrogen reduction reaction by gadolinium doping in 2D bismuth molybdate nanosheets. Appl. Sur. Sci..

[16] Islam M.H., Mehrabi H., Coridan R.H., Burheim O.S., Hihn J.Y., Pollet B.G. (2021). The effects of power ultrasound (24 kHz) on the electrochemical reduction of CO_2_ on polycrystalline copper electrodes. Ultrason. Sonochem..

[17] Zheng W.Z., Wang D.S., Zhang Y.K., Zheng S.X., Yang B., Li Z.J., Rodriguez R.D., Zhang T., Lei L.C., Yao S.Y., Hou Y. (2022). Promoting industrial-level CO_2_ electroreduction kinetics via accelerating proton feeding on a metal-free aerogel electrocatalyst. Nano Energy.

[18] Kiani M., Tian X.Q., Zhang W.X. (2021). Non-precious metal electrocatalysts design for oxygen reduction reaction in polymer electrolyte membrane fuel cells: recent advances, challenges and future perspectives. Coord. Chem. Rev..

[19] Zhang S., Zhang X., Rui Y., Wang R.H., Li X.J. (2021). Recent advances in non-precious metal electrocatalysts for pH-universal hydrogen evolution reaction, Green. Energy Environ..

[20] He W., Zhang R., Cao D.a., Li Y., Zhang J., Hao Q., Liu H., Zhao J., Xin H.L. (2023). Super-hydrophilic microporous Ni(OH)_x_/Ni_3_S_2_ heterostructure electrocatalyst for large-current-density hydrogen evolution. Small.

[21] Dessalle A., Quilez-Bermejo J., Fierro V., Xu F.N., Celzard A. (2022). Recent progress in the development of efficient biomass-based ORR electrocatalysts. Carbon.

[22] Liang T.T., Wang A.Q., Ma D.Q., Mao Z.P., Wang J., Xie J.P. (2022). Low-dimensional transition metal sulfide-based electrocatalysts for water electrolysis: overview and perspectives. Nanoscale.

[23] Feng M., Huang J.L., Peng Y., Huang C.R., Yue X., Huang S.M. (2022). Tuning the electronic structures of cobalt-molybdenum bimetallic carbides to boost the hydrogen oxidation reaction in alkaline medium. Chem. Eng. J..

[24] Li Y.J., Guo S.J. (2019). Nobel metal-based 1D and 2D electrocatalytic nanomaterials: recent progress, challenges and perspectives. Nano Today.

[25] Z. J. Li, L. Zhai, Y. Y. Ge, Z. Q. Huang, Z. Y. Shi, J. W. Liu, W. Zhai, J. Z. Liang, H. Zhang, Wet-chemical synthesis of 2D metal nanomaterials for electrocatalysis, Natl. Sci. Rev. 9 (2022) nwab142. 10.1093/nsr/ nwab142. 10.1093/nsr/nwab142 PMC9113131 35591920

[26] Peng J., Dong W., Wang Z., Meng Y., Liu W., Song P., Liu Z. (2021). Recent advances in 2D transition metal compounds for electrocatalytic full water splitting in neutral media. Mater. Today Adv..

[27] Jin X.Y., Piao H.Y., Sun Y.Y., Choy J.H., Hwang S.J. (2022). High efficiency of self-assembly between exfoliated MXene and layered-double-hydroxide nanosheets in exploring high-performance oxygen evolution reaction electrocatalysts, 2D. Mater..

[28] Wei Y.X., Wu D.K., Yong C.Y., Wang Z.N., Zhong P., Qiu J.J., Fan J.T., Sun J., Lei Y.M., Wu X.Q. (2022). Robust and highly conductive Ti4O7/MXene nanocomposites as high-performance and long cyclic stability oxygen reduction electrocatalysts. Appl. Sur. Sci..

[29] Wang Y., Nian Y., Biswas A.N., Li W., Han Y., Chen J.G.G. (2021). Challenges and opportunities in ultilizing MXenes of carbides and nitrides as electrocatalysts. Adv. Energy Mater..

[30] Tsounis C., Kumar P.V., Masood H., Kulkarni R.P., Gautam G.S., Muller C.R., Amal R., Kuznetsov D.A. (2023). Advancing MXene electrocatalysts for energy conversion reactions: surface, stoichiometry, and stability. Angew. Chem. Int. Ed..

[31] Yi H., Liu S.Y., Lai C., Zeng G.M., Li M.F., Liu X.G., Li B.S., Huo X.Q., Qin L., Li L., Zhang M.M., Fu Y.K., An Z.W., Chen L. (2021). Recent advance of transition-metal-based layered double hydroxide nanosheets: synthesis, properties, modification, and electrocatalytic applications. Adv. Energy Mater..

[32] Yu Q., Lv J.S., Li J.T., Yu R.H., Wu J.S., Xi S.B., Li X.Y., Xu N., Zhou L., Mai L.Q. (2022). ZIF-mediated anchoring of Co species on N-doped carbon nanorods as an efficient cathode catalyst for Zn-air batteries. Energy Environ. Mater..

[33] Zhang Y.Y., Kong X.P., Lin X.R., Hu K.L., Zhao W.W., Xie G.Q., Lin X., Lin X.J., Ito Y., Qiu H.J. (2021). Enhanced bifunctional catalytic activities of N-doped graphene by Ni in a 3D trimodal nanoporous nanotubular network and its ultralong cycling performance in Zn-air batteries. J. Energy Chem..

[34] Zhu L.L., Wang Z., Li C.D., Li H., Huang Y.A., Li H., Wu Z.Q., Lin S., Li N., Zhu X.B., Sun Y.P. (2022). Highly stable 1T-MoS_2_ by magneto-hydrothermal synthesis with Ru modification for efficient hydrogen evolution reaction. J. Mater. Chem. A.

[35] Qu J., Li Y., Li F., Li T.M., Wang X.Y., Yin Y., Ma L.B., Schmidt O.G., Zhu F. (2022). Direct thermal enhancement of hydrogen evolution reaction of on-chip monolayer MoS_2_. ACS Nano.

[36] Li Y., Gu Q.F., Johannessen B., Zheng Z., Li C., Luo Y.T., Zhang Z.Y., Zhang Q., Fan H.N., Luo W.B., Liu B.L., Dou S.X., Liu H.K. (2021). Synergistic Pt doping and phase conversion engineering in 2D MoS_2_ for efficient hydrogen evolution. Nano Energy.

[37] Zhao X.H., Levell Z.H., Yu S., Liu Y.Y. (2022). Atomistic understanding of 2D electrocatalysts from first principles. Chem. Rev..

[38] Jin X.Y., Jang H., Jarulertwathana N., Kim M.G., Hwang S.J. (2022). Atomically thin holey 2D Ru_2_P nanosheets for enhanced hydrogen evolution electrocatalysis. ACS Nano.

[39] Lyu X., Zhang W.N., Li G., Shi B.W., Zhang Y.N., Chen H., Li S.C., Wang X. (2020). 2D porous PtPd nanostructure electrocatalysts for oxygen reduction reaction. ACS Appl. Nano Mater..

[40] Xu X., Liang T., Kong D., Wang B., Zhi L. (2021). Strain engineering of 2D materials for advanced electrocatalysts, Mater. Today. Nano.

[41] Hasan M.M., Khedr G.E., Zakaria F., Allam N.K. (2022). Intermolecular electron transfer in electrochemically exfoliated BCN-Cu nanosheet electrocatalysts for efficient hydrogen evolution. ACS Appl. Energy Mater..

[42] Xu B.Y., Zhang Y., Li L.G., Shao Q., Huang X.Q. (2022). Recent progress in low-dimensional palladium-based nanostructures for electrocatalysis and beyond. Coord. Chem. Rev..

[43] Chemat F., Zill-e-Huma M.K.K. (2011). Applications of ultrasound in food technology: processing, preservation and extraction. Ultrason. Sonochem..

[44] Sricharoen P., Limchoowong N., Techwongstien S., Chanthai S. (2019). Ultrasound-assisted emulsification microextraction coupled with salt-induced demulsification based on solidified floating organic drop prior to HPLC determination of Sudan dyes in chili products. Arab. J. Chem..

[45] Bang J.H., Suslick K.S. (2010). Applications of ultrasound to the synthesis of nanostructured materials. Adv. Mater..

[46] Kumar P.R., Suryawanshi P.L., Gumfekar S.P., Sonawane S.H. (2017). Ultrasound-assisted synthesis of conducting polymer-based electrocatalysts for fuel cell applications. Chem. Eng. Process..

[47] Xu X.D., Zhang Y.L., Sun H.Y., Zhou J.W., Yang F., Li H., Chen H., Chen Y.C., Liu Z., Qiu Z.P., Wang D., Ma L.P., Wang J.W., Zeng Q.G., Peng Z.Q. (2021). Progress and perspective: MXene and MXene-based nanomaterials for high-performance energy storage devices. Adv. Electron. Mater..

[48] Wu Q., Ouyang J.J., Xie K.P., Sun L., Wang M.Y., Lin C.J. (2012). Ultrasound-assisted synthesis and visible-light-driven photocatalytic activity of Fe-incorporated TiO_2_ nanotube array photocatalysts. J. Hazard. Mater..

[49] Abdullah M.I., Hameed A., Zhang N., Ma M.M. (2019). Nickel nanocrystal assemblies as efficient electrocatalysts for hydrogen evolution from pH-neutral aqueous solution. ChemElectroChem.

[50] Al-Hamadani Y.A.J., Jun B.M., Yoon M., Taheri-Qazvini N., Snyder S.A., Jang M., Yoon Y. (2020). Applications of MXene-based membranes in water purification: a review. Chemmsphere.

[51] Chen Q.T., Yang M.S., Chen F.H., Zhang Z.Q., Liang W.W., Shi X.D., Zhang Y.H., Jiang L.Y., Fang S.M. (2023). Synthesis of Co_4_S_3_/Co_9_S_8_ nanosheets and their Fe/Cr dual heteroatom co-doped components for the promoted OER properties. J. Solid State Electrochem..

[52] Zheng J.X., Guo Y.L., Zhu L.X., Deng H.L., Shang Y.J. (2021). Cavitation effect in 2D ultrasonic rolling process. Ultrasonics.

[53] Z. W. Li, Z. W. Xu, L. Ma, S. Wang, X. S. Liu, J. C. Yan, Cavitation at filler metal/substrate interface during ultrasonic-assisted soldering. Part 2: cavitation erosion effect, Ultrason. Sonochem. 50 (2018) 278-288. 10.1016/j.ultsonch. 2018.09.027. 10.1016/j.ultsonch.2018.09.027 30274890

[54] Ye L.Z., Zhu X.J., Liu Y. (2019). Numerical study on dual-frequency ultrasonic enhancing cavitation effect based on bubble dynamic evolution. Ultrason. Sonochem..

[55] Tan K.L., Yeo S.H. (2019). Bubble dynamics and cavitation intensity in milli-scale channels under an ultrasonic horn. Ultrason. Sonochem..

[56] Liu Z.W., Ji C.H., Wang B., Sun S.Q. (2019). Role of a nanoparticle on ultrasonic cavitation in nanofluids, Micro. Nano Lett..

[57] Silva L.I., Mirabella D.A., Tomba J.P., Riccardi C.C. (2019). Optimizing graphene production in ultrasonic devices. Ultrasonics.

[58] Harvey E.N., McElroy W.D., Whiteley A.H. (1947). On cavity formation in water. J. Appl. Phys.

[59] Lascaud J., Parodi K. (2023). On the potential biological impact of radiation-induced acoustic emissions during ultra-high dose rate electron radiotherapy: a preliminary study. Phys. Med. Biol..

[60] Bussonniere A., Liu Q.X., Tsai P.A. (2020). Cavitation nuclei regeneration in a water-particle suspension. Phys. Rev. Lett..

[61] Bremond N., Arora M., Ohl C.D., Lohse D. (2005). Cavitation on surfaces. J. Phys. Condens. Mat..

[62] Bremond N., Arora M., Ohl C.D., Lohse D. (2006). Controlled multibubble surface cavitation. Phys. Rev. Lett..

[63] Nguyen T.T., Asakura Y., Okada N., Koda S., Yasuda K. (2017). Effect of ultrasonic cavitation on measurement of sound pressure using hydrophone. Jpn. J. Appl. Phys..

[64] Silva F.V., Zanardi M.A., de Souza T.M. (2021). Analytical-numerical modeling of journal bearings with non-Newtonian fluids and cavitation effects. J. Braz. Soc. Mech. Sci. Eng..

[65] Liu H.X., Deng Z., Chen J., Kang C., Li B. (2021). Synergetic effect of corrosion and ultrasonic cavitation erosion on leaded brass. J. Mater. Eng. Perform..

[66] Yang R., Huang N.L., Tian Y., Qin J.H., Lu P.F., Chen H., Li H., Chen X.Y. (2022). Insights into the exceptional cavitation erosion resistance of laser surface melted Ni-WC composites: the effects of WC morphology and distribution. Surf. Coat. Tech..

[67] Chen F.J., Du J.H., Zhou S.Z. (2020). Cavitation erosion behaviour of incoloy alloy 865 in NaCl solution using ultrasonic vibration. J. Alloys Compd..

[68] Huang H.J., Shu D., Fu Y.A., Wang J., Sun B.D. (2014). Synchrotron radiation X-ray imaging of cavitation bubbles in Al-Cu alloy melt. Ultrason. Sonochem..

[69] Hasan M., Meidani A.R.N. (2009). Ultrasonic treatment of a solidifying Al-Cu melt in the presence of micron-sized hydrogen bubbles. Light Metals.

[70] Doktycz S.J., Suslick K.S. (1990). Interparticle collisions driven by ultrasound. Science.

[71] Prozorov T., Prozorov R., Suslick K.S. (2004). High velocity interparticle collisions driven by ultrasound. J. Am. Chem. Soc..

[72] Xu H.X., Zeiger B.W., Suslick K.S. (2013). Sonochemical synthesis of nanomaterials. Chem. Soc. Rev..

[73] Guittonneau F., Abdelouas A., Grambow B., Huclier S. (2010). The effect of high power ultrasound on an aqueous suspension of graphite. Ultrason. Sonochem..

[74] Hernandez Y., Nicolosi V., Lotya M., Blighe F.M., Sun Z., De S., McGovern I.T., Holland B., Byrne M., Gun'Ko Y.K., Boland J.J., Niraj P., Duesberg G., Krishnamurthy S., Goodhue R., Hutchison J., Scardaci V., Ferrari A.C., Coleman J.N. (2008). High-yield production of graphene by liquid-phase exfoliation of graphite. Nat. Nanotechnol..

[75] Li Q.Y., Zhang X., Wu G.Z., Xu S., Wu C.F. (2008). Sonochemical preparation of carbon nanosheet from carbon black. Ultrason. Sonochem..

[76] Kass M.D. (2000). Ultrasonically induced fragmentation and strain in alumina particles. Mater. Lett..

[77] Gopi K.R., Nagarajan R. (2008). Advances in nanoalumina ceramic particle fabrication using sonofragmentation. IEEE Trans. Nanotechnol..

[78] Teipel U., Leisinger K., Mikonsaari I. (2004). Comminution of crystalline material by ultrasonics. Int. J. Miner. Process..

[79] Zeiger B.W., Suslick K.S. (2011). Sonofragmentation of molecular crystals. J. Am. Chem. Soc..

[80] Ke C.M., Huang J.W., Liu S. (2021). 2D ferroelectric metal for electrocatalysis. Mater. Horiz..

[81] Zhong H.X., Wang M.C., Chen G.B., Dong R.H., Feng X.L. (2022). 2D conjugated metal-organic frameworks for electrocatalysis: opportunities and challenges. ACS Nano.

[82] Zhao X.N., Zhou X.L., Yang S.Y., Min Y., Chen J.J., Liu X.W. (2022). Plasmonic imaging of the layer-dependent electrocatalytic activity of 2D catalysts. Nat. Commun..

[83] Wei Y., Soomro R.A., Xie X.Q., Xu B. (2021). Design of efficient electrocatalysts for hydrogen evolution reaction based on 2D MXenes. J. Energy Chem..

[84] Lai Q.X., Zheng H.M., Tang Z.M., Bi D., Chen N.N., Liu X.J., Zheng J., Liang Y.Y. (2021). Balance of N-doping engineering and carbon chemistry to expose edge graphitic N sites for enhanced oxygen reduction electrocatalysis. ACS Appl. Mater. Interfaces.

[85] Doan T.L.L., Nguyen D.C., Prabhakaran S., Kim D., Tran D.T., Kim N.H., Lee J.H. (2021). Single-atom co-decorated MoS_2_ nanosheets assembled on metal nitride nanorod arrays as an efficient bifunctional electrocatalyst for pH-universal water splitting. Adv. Funct. Mater..

[86] Malali P., Muchharla B., Sadasivuni K.K., Cao W., Elsayed H.E., Adedeji A., Karoui A., Abdullah A.M., Spurgeon J.M., Kumar B. (2022). Low platinum-loaded molybdenum co-catalyst for the hydrogen evolution reaction in alkaline and acidic media. Langmuir.

[87] Cao Q., Hao S., Wu Y.W., Pei K., You W.B., Che R.C. (2021). Interfacial charge redistribution in interconnected network of Ni_2_P-Co_2_P boosting electrocatalytic hydrogen evolution in both acidic and alkaline conditions. Chem. Eng. J..

[88] Hu Q., Zhang S.T., Li W.P., Xiong J.L., Zou X.F., Shen H.J. (2022). Regulating the structure and morphology of nickel sulfides for electrochemical energy storage: the role of solvent pH. Chem. Eng. J..

[89] Jian C.Y., Hong W.T., Cai Q., Li J., Liu W. (2020). Surface electron state engineering enhanced hydrogen evolution of hierarchical molybdenum disulfide in acidic and alkaline media. Appl. Catal. B Environ..

[90] Shen Q., Jiang L., Miao J., Hou W., Zhu J.-J. (2008). Sonoelectrochemical synthesis of CdSe nanotubes. Chem. Commun..

[91] Jiang L.P., Xu S., Miao J.J., Wang H., Zhu J.J. (2006). Sonochemical synthesis of US and CdSe nanowires. J. Nanosci. Nanotechnol..

[92] Das A., Wai C.M. (2014). Ultrasound-assisted synthesis of PbS quantum dots stabilized by 1,2-benzenedimethanethiol and attachment to single-walled carbon nanotubes. Ultrason. Sonochem..

[93] Nehru R., Hsu Y.F., Wang S.F., Chen C.W., Dong C.D. (2021). Selective electrochemical sensing platform based on the synergy between carbon black and single-crystalline bismuth sulfide for rapid analysis of antipyretic drugs. ACS Appl. Bio Mater..

[94] Bogolubsky A.V., Moroz Y.S., Mykhailiuk P.K., Ostapchuk E.N., Rudnichenko A.V., Dmytriv Y.V., Bondar A.N., Zaporozhets O.A., Pipko S.E., Doroschuk R.A., Babichenko L.N., Konovets A.I., Tolmachev A. (2015). One-pot parallel synthesis of alkyl sulfides, sulfoxides, and sulfones. ACS Comb. Sci..

[95] Ghows N., Entezari M.H. (2011). A novel method for the synthesis of CdS nanoparticles without surfactant. Ultrason. Sonochem..

[96] Hinnemann B., Moses P.G., Bonde J., Jorgensen K.P., Nielsen J.H., Horch S., Chorkendorff I., Norskov J.K. (2005). Biornimetic hydrogen evolution: MoS_2_ nanoparticles as catalyst for hydrogen evolution. J. Am. Chem. Soc..

[97] Jaramillo T.F., Jorgensen K.P., Bonde J., Nielsen J.H., Horch S., Chorkendorff I. (2007). Identification of active edge sites for electrochemical H_2_ evolution from MoS_2_ nanocatalysts. Science.

[98] Yadav A.A., Hunge Y.M., Kang S.W. (2021). Ultrasound assisted synthesis of highly active nanoflower-like CoMoS_4_ electrocatalyst for oxygen and hydrogen evolution reactions. Ultrason. Sonochem..

[99] Vinoth S., Govindasamy M., Wang S.F., Anandaraj S. (2020). Layered nanocomposite of zinc sulfide covered reduced graphene oxide and their implications for electrocatalytic applications. Ultrason. Sonochem..

[100] Yang G.Z., Meng M., Wang X.Q., Peng C.X., Xue Y.H., Yang J.H., Tang Z.H. (2022). Three-dimensional crumpled reduced graphene oxide/Co_9_S_8_ nanocomposites as efficient electrocatalyst for oxygen evolution reaction. J. Alloys Compd..

[101] Xu L.N., Zhang Y.H., Feng L.L., Li X., An Q. (2021). A facile preparation method for MoS_2_ nanosheets and their well-controllable interfacial assembly with PEDOT: PSS for effective electrochemical hydrogen evolution reactions. J. Mater. Sci..

[102] Yu J.F., Wang Q., O’Hare D., Sun L.Y. (2017). Preparation of two dimensional layered double hydroxide nanosheets and their applications. Chem. Soc. Rev..

[103] Wang Y.Y., Yan D.F., EIhankari S., Zou Y.Q., Wang S.Y. (2018). Recent progress on layered double hydroxides and their derivatives for electrocatalytic water splitting. Adv. Sci..

[104] Silva A.F., Duarte J.L., Meili L. (2021). Different routes for MgFe/LDH synthesis and application to remove pollutants of emerging concern. Sep. Purif. Technol..

[105] Belviso C., Piancastelli A., Sturini M., Belviso S. (2020). Synthesis of composite zeolite-layered double hydroxides using ultrasonic neutralized red mud. Microporous Mesoporous Mater..

[106] Yang Y.J., Li W.K. (2018). Ultrasonic assisted coating of multiwalled carbon nanotubes with NiFe layered double hydroxide for improved electrocatalytic oxygen reduction. J. Electroanal. Chem..

[107] Koshikawa H., Murase H., Hayashi T., Nakajima K., Mashiko H., Shiraishi S., Tsuji Y. (2020). Single nanometer-sized NiFe-layered double hydroxides as anode catalyst in anion exchange membrane water electrolysis cell with energy conversion efficiency of 74.7% at 1.0 A cm^-2^. ACS Catal..

[108] Hameed A., Zulfiqar F., Iqbal W., Ali H., Shah S.S.A., Nadeem M.A. (2022). Electrocatalytic water oxidation on CuO-Cu_2_O modulated cobalt-manganese layered double hydroxide. RSC Adv..

[109] Wang Y.Y., Qiao M., Li Y.F., Wang S.Y. (2018). Tuning surface electronic configuration of NiFe LDHs nanosheets by introducing cation vacancies (Fe or Ni) as highly efficient electrocatalysts for oxygen evolution reaction. Small.

[110] Feng X.T., Jiao Q.Z., Chen W.X., Dang Y.L., Dai Z., Suib S.L., Zhang J.T., Zhao Y., Li H.S., Feng C.H. (2021). Cactus-like NiCo_2_S_4_@NiFe LDH hollow spheres as an effective oxygen bifunctional electrocatalyst in alkaline solution. Appl. Catal. B Environ..

[111] Jeghan S., Kim N., Lee G. (2021). Mo-incorported three-dimensional hierarchical ternary nickel-cobalt-molybdenum layer double hydroxide for high-efficiency water splitting. Int. J. Hydrogen Energy.

[112] Keyikoglu R., Khataee A., Lin H., Orooji Y. (2022). Vanadium (V)-doped ZnFe layered double hydroxide for enhanced sonocatalytic degradation of pymetrozine. Chem. Eng. J..

[113] Keyikoglu R., Dogan I., Khataee A., Orooji Y., Kobya M., Yoon Y. (2022). Synthesis of visible responsive ZnCoFe layered double hydroxide towards enhanced photocatalytic activity in water treatment. Chemosphere.

[114] Keyikoglu R., Khataee A., Orooji Y., Kobya M. (2022). Synergistic effect of Fe and Co metals for the enhanced activation of hydrogen peroxide in the heterogeneous electro-Fenton process by Co-doped ZnFe layered double hydroxide. J. Environ. Chem. Eng..

[115] Wang T.J., Liu X.Y., Li Y., Li F.M., Deng Z.W., Chen Y. (2020). Ultrasonication-assisted and gram-scale synthesis of Co-LDH nanosheet aggregates for oxygen evolution reaction. Nano Res..

[116] Munonde T.S., Zheng H.T., Nomngongo P.N. (2019). Ultrasonic exfoliation of NiFe LDH/CB nanosheets for enhanced oxygen evolution catalysis. Ultrason. Sonochem..

[117] Li X.M., Hao X.G., Wang Z.D., Abudula A., Guan G.Q. (2017). In-situ intercalation of NiFe LDH materials: an efficient approach to improve electrocatalytic activity and stability for water splitting. J. Power Sources.

[118] Liu X.R., Shi J.M., Bai X.F., Wu W. (2021). Ultrasound-excited hydrogen radical from NiFe layered double hydroxide for preparation of ultrafine supported Ru nanocatalysts in hydrogen storage of N-ethylcarbazole. Ultrason. Sonochem..

[119] Foruzin L.J., Rezvani Z. (2020). Ultrasonication construction of the nano-petal NiCoFe-layered double hydroxide: an excellent water oxidation electrocatalyst. Ultrason. Sonochem..

[120] Jia X.D., Zhang X., Zhao J.Q., Zhao Y.F., Zhao Y.X., Waterhouse G., Shi R., Wu L., Tung C., Zhang T. (2019). Ultrafine monolayer Co-containing layered double hydroxide nanosheets for water oxidation. J. Energy Chem..

[121] Wu B., Meng H.B., Morales D.M., Zeng F., Zhu J.J., Wang B., Risch M., Xu Z.C., Petit T. (2022). Nitrogen-rich carbonaceous materials for advanced oxygen electrocatalysis: synthesis, characterization, and activity of nitrogen sites. Adv. Funct. Mater..

[122] Wang J., Kong H., Zhang J.Y., Hao Y., Shao Z.P., Ciucci F. (2021). Carbon-based electrocatalysts for sustainable energy applications. Prog. Mater. Sci..

[123] Bouleau L., Perez-Rodriguez S., Quilez-Bermejo J., Izquierdo M.T., Xu F., Fierro V., Celzard A. (2022). Best practices for ORR performance evaluation of metal-free porous carbon electrocatalysts. Carbon.

[124] Sebastian N., Yu W.C., Hu Y.C., Balram D., Yu Y.H. (2019). Sonochemical synthesis of iron-graphene oxide/honeycomb-like ZnO ternary nanohybrids for sensitive electrochemical detection of antipsychotic drug chlorpromazine. Ultrason. Sonochem..

[125] Zhang M.L., Tao H.C., Liu Y.C., Yan C., Hong S., Masa J., Robertson A.W., Liu S.Z., Qiu J.S., Sun Z.Y. (2019). Ultrasound-assisted nitrogen and boron cooping of graphene oxide for efficient oxygen reduction reaction. ACS Sustainable Chem. Eng..

[126] Ding W., Sun M.X., Zhang Z.H., Lin X.J., Gao B.W. (2020). Ultrasound-promoted synthesis of γ-graphene for supercapacitor and photoelectrochemical applications. Ultrason. Sonochem..

[127] Anandan S., Manivel A., Ashokkumar M. (2012). One-step sonochemical synthesis of reduced graphene oxide/Pt/Sn hybrid materials and their electrochemical properties. Full Cells.

[128] Rani K.K., Karuppiah C., Wang S., Alaswad S.O., Sireesha P., Devasenathipathy R., Jose R., Yang C.C. (2020). Direct pyrolysis and ultrasound assisted preparation of N, S co-doped graphene/F_e_3C nanocomposite as an efficient electrocatalyst for oxygen reduction and oxygen evolution reactions. Ultrason. Sonochem..

[129] Shi C.W., Liu Y.H., Qi R.Y., Li J.T., Zhu J.X., Yu R.H., Li S.D., Hong X.F., Wu J.S., Xi S.B., Zhou L., Mai L.Q. (2021). Hierarchical N-doped carbon spheres anchored with cobalt nanocrystals and single atoms for oxygen reduction reaction. Nano Energy.

[130] Vivekanandan A.K., Subash V., Chen S.M. (2020). Sonochemical synthesis of nickel-manganous oxide nanocrumbs decorated partially reduced graphene oxide for efficient electrochemical reduction of metronidazole. Ultrason. Sonochem..

[131] Wang H.H., Li L., Sheng S.Z., Wang C.N., Qu T.P., Hou D., Wang D.Y., Sheng M.Q. (2022). Synthesis of low-cost Co-Sn-Pd/rGO catalysts via ultrasonic irradiation and their electrocatalytic activities toward oxygen reduction reaction. Can. J. Chem. Eng..

[132] Cui Z.L., Bai X.F. (2021). Ultrasonic-assisted synthesis of two dimensional coral-like Pd nanosheets supported on reduced graphene oxide for enhanced electrocatalytic performance. Ultrason. Sonochem..

[133] Zhang X.Y., Zhang W., Zhao H.T. (2022). Electrochemical performance of Ti_3_C_2_T_x_ MXenes obtained via ultrasound assisted LiF-HCl method. Mater. Today Commun..

[134] Zhang W., Zhang X.Y. (2022). The effect of ultrasound on synthesis and energy storage mechanism of Ti_3_C_2_T_x_ MXene. Ultrason. Sonochem..

[135] Liu W.Z., Sun M.X., Ding Z.P., Gao B.W., Ding W. (2021). Ti_3_C_2_ MXene embellished g-C_3_N_4_ nanosheets for improving photocatalytic redox capacity. J. Alloys Compd..

[136] Zhang X.Y., Zhang W., Zhao H.T. (2022). Ultrasound-assisted fabrication of Ti_3_C_2_T_x_ MXene toward enhanced energy storage performance. Ultrason. Sonochem..

[137] Han M.N., Yang J., Jiang J.T., Jing R.W., Ren S.J., Yan C. (2021). Efficient tuning the electronic structure of N-doped Ti-based MXene to enhance hydrogen evolution reaction. J. Colloid Interface Sci..

[138] Zhou Y., Wu Y.S., Guo D.X., Li J.L., Dong G.H., Chai D.F., Yang X., Fu S.S., Sui G.Z. (2023). Surfur vacancy modulated nickel-doped Co_4_S_3_ hollow nanocube/nitrogen-doped V_2_CT_x_ MXene nanosheet composites for optimizing the hydrogen evolution reaction. Mater. Chem. Front..

[139] Deng L.M., Hu F., Ma M.Y., Huang S.C., Xiong Y.X., Chen H.Y., Li L.L., Peng S.J. (2021). Electronic modulation of metal-organic frameworks by interfacial bridging for efficient pH-universal hydrogen evolution. Angew. Chem. Int. Ed..

